# FITNESS AND TIME OF MUTATION ACQUISITION A BETTER PREDICTOR OF OUTCOME IN CLONAL HEMOPOIESIS COMPARED TO CLONE SIZE

**DOI:** 10.1093/geroni/igae098.1448

**Published:** 2024-12-31

**Authors:** Kristina Kirschner, Simon Cox, Tamir Chandra Neil Robertson Eric Crespo, Linus Schumacher

**Affiliations:** Mayo Clinic, Rochester, Minnesota, United States; University of Edinburgh, Edinburgh, Scotland, United Kingdom

## Abstract

The prevalence of clonal hematopoiesis of indeterminate potential (CHIP) in healthy individuals increases rapidly from age 60 onwards and has been associated with increased risk for malignancy, heart disease and ischemic stroke. CHIP is driven by somatic mutations in stem cells that are also drivers of myeloid malignancies. We previously reported gene specific fitness effects in two longitudinal cohorts of aging. We now set out to define fitness effects across three longitudinal cohorts of aging in a total of 713 participants. We found that fitness correlates with timing of mutations, which needs to be taken into account when predicting health outcomes. We show that fitness and the time of mutations are superior predictors of outcomes such as all-cause mortality compared to the commonly used clone size (variant allele frequency). Our new predictor even outperforms age, the most important predictor for the onset of age-related disease thus far. In conclusion, the fitness landscape of mutations and their context in terms of clonal composition and timing of mutation acquisition are important parameters to bring CH predictors into the clinic.

